# Passive anti-amyloid immunotherapy in Alzheimer's disease: What are the most promising targets?

**DOI:** 10.1186/1742-4933-10-18

**Published:** 2013-05-11

**Authors:** Jens Moreth, Chrystelle Mavoungou, Katharina Schindowski

**Affiliations:** 1 Institute of Applied Biotechnology, Faculty for Biotechnology, Biberach University of Applied Science, Karlstrasse 11, Biberach/Riss, D-88400, Germany

**Keywords:** Passive immunization, Dementia, Therapeutic antibodies, Effector function, Oligomers, ADDLs, Protofibrils, Regulatory strategy

## Abstract

Alzheimer’s disease (AD) is the most common dementia in the industrialized world, with prevalence rates well over 30% in the over 80-years-old population. The dementia causes enormous costs to the social healthcare systems, as well as personal tragedies for the patients, families and caregivers. AD is strongly associated with Amyloid-beta (Aβ) protein aggregation, which results in extracellular plaques in the brain, and according to the amyloid cascade hypothesis appeared to be a promising target for the development of AD therapeutics. Within the past decade convincing data has arisen positioning the soluble prefibrillar Aβ-aggregates as the prime toxic agents in AD. However, different Aβ aggregate species are described but their remarkable metastability hampers the identification of a target species for immunization. Passive immunotherapy with monoclonal antibodies (mAbs) against Aβ is in late clinical development but recently the two most advanced mAbs, Bapineuzumab and Solanezumab, targeting an N-terminal or central epitope, respectively, failed to meet their target of improving or stabilizing cognition and function. Preliminary data from off-label treatment of a small cohort for 3 years with intravenous polyclonal immunoglobulins (IVIG) that appear to target different conformational epitopes indicate a cognitive stabilization. Thus, it might be the more promising strategy reducing the whole spectrum of Aβ-aggregates than to focus on a single aggregate species for immunization.

## Aß-aggregates and their impact in choosing the right antibody

Alzheimer's disease (AD) is the most common form of dementia. It accounts for 60-70% of all cases among the oldest old [1]; and countries in demographic transition will experience the greatest growth. AD is a multifactorial disease with pathogenic cerebral protein aggregation, including aggregation of hyperphosphorylated tau (phospho-tau) and the aggregation and deposition of Amyloid-β (Aβ), accompanied by oxidative stress and glial activation [2]. Thus, many pathophysiological pathways coexist, resulting in synaptic dysfunction and severe neuronal loss that cause deterioration and finally loss of memory and cognition. Within the past two decades substantial efforts have been made to elucidate the toxic nature of Aβ in AD. The primary event that induces the abnormal accumulation of Aβ is the dysregulated proteolytic processing by secretases of its parent molecule, the amyloid precursor protein (APP) [3]. Dysregulated APP-processing results in the Aβ-peptide of predominantly 39 to 43 residues, but even smaller species occur. Further post-translational modifications result in a various number of N- and C-terminal variants of the Aβ-peptide [4], increasing heterogeneity and, thus, the number of possible targets.

The aggregation of Aβ species is thought to play a pivotal role in the disease progression of AD through a cascade of events, called the amyloid cascade hypothesis [5-7]. In the light of the recent clinical trials with anti-Aβ drugs the amyloid cascade hypothesis is again a subject of discussion.

**Figure 1:**
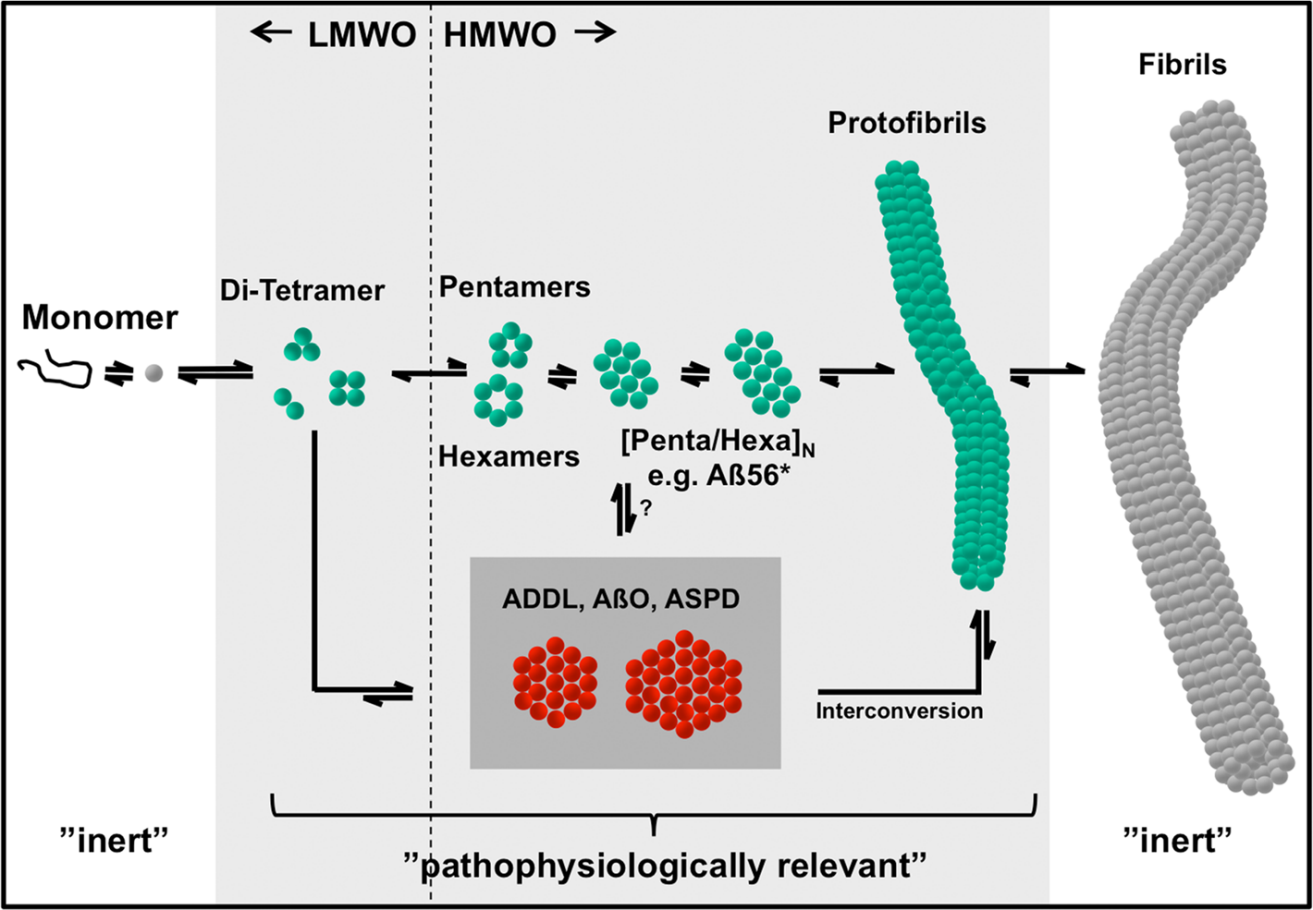
**Pathways of aggregation and observed Aß-aggregate intermediates. **Monomeric Aß folds to the activated state and then exists in rapid equilibrium with low molecular weight oligomers, which aggregate over various transient high molecular weight intermediates to matured fibrils. The definition of LMW and HMW oligomers is related to the elution profile of Aß-aggregates in size exclusion chromatography, revealing two predominant peaks at the exclusion limit (>60 kDa) and at the void volume (4-20 kDa), respectively. The HMW intermediates comprise pentamers, hexamers and multiples thereof, finally forming protofibrils, which are the precursors for multi-stranded ribbons of matured fibrils. Further neurotoxic aggregate species e.g. AßO, ADDL and ASPD are believed to aggregate over alternative pathways but preliminary data revealed that these are able to converge into the other pathways of aggregation (inter-conversion). Interestingly, every change in the experimental paradigm can provoke this aggregate conversion. Therefore, one might assume that many different aggregates coexist and, thus, neurotoxicity can be attributed to several pathogenic modes of action. Monomers and fibrils are believed to be biologically inert; however fibrils are able to collapse into protofibrils and then also reveal toxicity. The broad range of prefibrillar aggregates have been reported as pathophysiologically relevant in AD.

The self-association of Aβ-peptide results in aggregates with varying morphology and molecular weight (see Figure 1). After sequestration of the nascent monomeric Aβ-peptide subsequent to secretase cleavage Aß folds to an activated monomeric state and then exists in rapid equilibrium with low molecular weight aggregates [8]. These further associate over various transient intermediates to mature insoluble Aβ-fibrils, which accumulate in the AD brain as senile plaques. Further investigations focused on the prefibrillar aggregates, the water-soluble oligomers, which are increased in AD-patients [9]. However, the term ”soluble” roughly describes aggregates, which remain in solution upon centrifugation at 15000 × g [10]. Monomeric and fibrillar Aβ are believed to be biologically inert; however Aß-fibrils can collapse back into protofibrils in the presence of lipids and then also reveal toxicity [11]. The oligomeric species show, so far, the best correlation to neuro-psychiatric analysis and synapse loss [12-14]. These results increased the impact of soluble, premature Aβ-aggregates in the disease progression of AD, which has been positioned in the reformulated amyloid cascade hypothesis by Selkoe and Walsh [15]. Huge efforts have been made to identify distinct Aβ-aggregates derived from synthetic peptide and natural sources, resulting in a plethora of described Aβ-species with overlapping size and morphology [16]: the Aβ-dimer [17,18], low-molecular weight oligomers, comprising dimeric to tetrameric Aß [19], pentamers and hexamers [20], the dodecameric Aß56* derived from transgenic mice and human brain [21,22], globulomers [23], Aß-oligomers [24], Alzheimer-derived diffusible ligands – ADDL [10], protofibrils [25], amylospheroids [26] just to mention the major Aβ-preparations in the field. A further complexity comes with the various Aβ-fragments including the N- and C-terminal truncated Aβ-species.

Some of these different Aβ-preparations have been used for immunization and screening to generate therapeutic monoclonal antibodies (mAbs) which are being evaluated in preclinical and clinical trials. Literally, the definitions for all the above-mentioned Aβ-aggregate species are based on the protocols for Aβ-aggregate preparation and the methods used for characterization. These definitions are not strictly used – resulting in controversy regarding the reported Aβ-species and the observed patho-physiological effects. It still remains unsolved whether the reported synthetic Aβ-aggregates exist *in vivo* since they are hardly comparable to the naturally derived Aβ-species. Synthetic Aβ allows for in-depth biophysical characterization based on high protein concentrations and purity however these Aβ-aggregates have to be critically reviewed since high protein concentrations are necessary for their generation. Intriguingly, endogenous Aβ reveals nanomolar concentrations in the brain and comprises a heterogeneous peptide-mixture with post-translational modifications and truncations at the N- and C-terminus, respectively. Furthermore, the characterization of natural derived Aβ, e.g. from tissue, CSF or blood, needs sophisticated methods for extraction which have an intrinsic effect on the identity of the Aβ-species and thus eludes a characterization of the aggregates originally present under native conditions. The common methods used for characterization of endogenous Aβ, e.g. SDS-PAGE, do not resolve the actual aggregative state. Thus, despite the reported presence of prefibrillar Aβ [27] the debate about the most relevant Aβ-species is still controversial. The identification of endogenous Aβ-aggregates is hampered owing to the dynamic and non-linear nature of aggregation and methodological limitations [28-30]. To this date the larger aggregates (e.g. ADDL, AβO) could not be shown *in vivo* by means of biophysics to structurally relate them to endogenous Aβ. Moreover, owing to the meta-stability and the ability for inter-conversion of different aggregation pathways, it is questionable whether to focus on a single “most-toxic” Aβ-species rather than the whole spectrum of Aβ-aggregates [28]. With regard to an anti-Aβ therapy, depleting total Aβ including all various Aβ fragments and aggregative species might be favorable compared to one conformation or species-specific antibodies since these might pick only one rogue out of many.

## Aβ immunotherapy with monoclonal antibodies

Although AD has been known about for over 100 years, there is still only symptomatic treatment available on the market. According to the amyloid cascade hypothesis, eradication of Aβ appears to be the aim for any disease-modifying therapy against AD, a therapy that is desperately needed to deal with the millions of AD patients in the upcoming decades.

The first active immunotherapy trial (AN-1792) using aggregated, full-length Aβ_42_ was halted after 6% of the patients developed severe meningoencephalitis [31]. The active vaccination with Aβ_42_ and adjuvant produced both a humoral and a cellular response against Aβ resulting in a strong and in some cases fatal immune response against the endogenous peptide Aβ [32].

Consequently, passive immunization as alternative was considered safer and more controllable than active immunization (see Table 1 for current trials). The mechanism of action of mAbs is firstly the capture of the target and secondly the effector function linked to the Fc domain of the mAb (for review see [33]). But how can antibodies against Aβ interfere with AD pathology? Despite the rapid advance of this therapeutic strategy into clinical trials and the hundreds of research papers, there still remain enigmatic aspects in Aβ immunotherapy [2]. Most importantly the mechanism of action is still not elucidated in rodents, let alone in humans, although many hypotheses have been proposed – including microglia-mediated phagocytosis, antibody-mediated alterations of Aβ aggregation and neutralization of Aβ toxicity, intracerebral sequestration of Aβ in a monomeric state and peripheral sink [34].

**Table 1:** **Passive immunotherapy for AD in clinical Phases, adopted from**[33]**, anti-Aβ antibodies in clinical Phases I - III**

**mAb**	**Specific for**	**Clinical trials**	**References**
**Bapineuzumab**, humanized 3D6	N-terminus (aa 1-5)	Phase III: trials were halted after completion of two trials demonstrated a failure to meet primary outcome measures of cognition and activities of daily living	[41-43]
**Solanezumab**, humanized m266	central (aa 16-24), accessible only on soluble Aβ	Phase III: ongoing as preventive trial in familial AD (DIAN). Trials failed to meet their primary endpoints in cognition and activities of daily living. A subsequent analysis of mild AD patients pooled from both trials showed a significant effect on cognition.	[44-46]
**Gantenerumab**, full human mAb	N-terminal (aa 3-12) and C-terminus (aa 18-27)	Phase III: ongoing in prodromal AD patients (DIAN), amyloid reduction but also ARIAs were observed in Phase I.	[47,48]
IVIG containing polyclonal NAbs-Aβ: **Gammagard**, **Octagam**, **New Gam**, **Flebogamma**	most NAbs-Aβ bind central and C-terminus as well as pathogenic conformations of Aβ (focus on dimers)	Phase III (Gammagard): ongoing, (improved cerebral glucose metabolism and cognitive stabilization of AD symptoms was shown in small clinical studies, too small for statistical evaluation)	[49-51]
		Phase III (Plasmapheresis with infusion of 20% albumin and Flebogamma): ongoing	
		Phase II (Octagam): cognition endpoints not met, but improved cerebral glucose metabolism	
		Phase II (NewGam): ongoing	
**Crenezumab**, humanized mMABT	conformational epitopes including oligomeric and protofibrillar forms, (aa 13-14 appears relevant)	Phase II: ongoing as long-term safety extension study.	[52]
		Preventive trial in an extended family carrying a *presenilin-1* mutation, which causes early onset AD planned for 2013.	
**BAN2401**, humanized mAb158	binds large-size Aβ protofibrils (>100 kDa)	Phase II: ongoing	[53,54]
**GSK933776**	N-terminus of Aβ	Phase I: two clinical trials for AD are completed and one for macular degeneration is ongoing. Further development for macular degeneration is in Phase II.	[55]
**AAB-003**, Fc-engineered Bapineuzumab	N-terminal (aa 1-5)	Phase I: ongoing. Lower toxicity (ARIAs) compared to Bapineuzumab is expected. Continuation as open-label extension study	[56]
**SAR228810**, humanized mAb 13C3	protofibrils, and low molecular weight Aβ	Phase I: ongoing	[57]
**BIIB037/BART**, full human IgG_1_	binds insoluble fibrillar human Aβ	Phase I: ongoing in prodromal AD patients	[58,59]

(*aa*, amino acid; *Aβ*, Amyloid-β; *ApoE4*, ApolipoproteinE4; *ARIA*, amyloid-related imaging abnormalities; *DIAN*, Dominantly Inherited Alzheimer Network; *IVIG*, Intravenous Immunoglobulin; *NAbs-Aβ*, natural occurring polyclonal Anti-Aβ antibodies).

Besides the microglial engulfment of Aβ, the other discussed mechanisms of action rely on binding Aβ and do not need effector function to clear Aβ. However, the first-in-class mAbs against Aβ are full IgG_1_s that strongly mediate pro-inflammatory effector functions. But, the Fc-domain of the antibodies can mediate toxicity since Aβ is also deposited in cerebral vasculature forming vascular plaques. In particular *ApoE4* (a hereditary risk factor for AD) carriers reveal vascular amyloid plaques [35]. Anti-Aβ antibodies capable to induce the complement system can lead to the formation of membrane attack complexes and thereby microbleedings i.e. microhemorrhages, vasogenic edema or if diagnosed with magnetic resonance imaging amyloid-related imaging abnormalities (ARIA) [36].

Conversely, many conflicting data exists about the mechanism of action of different epitope-specific antibodies and also about their blood-brain barrier passage (for review see [33]). Thus, one might assume that more than a single process takes place in passive Aβ immunotherapy. In summary, it appears that clearing cerebral Aβ, quite irrespective from the mechanism of action, is needed for improvement in brain pathology, synaptic transmission and cognition in AD animal models – given the well-known limitation of AD models.

The most advanced clinical candidates all recognize more or less linear epitopes on the Aβ peptide. Bapineuzumab (3D6) recognizes the linear N-terminus of Aβ and binds all forms of Aβ (e.g. prefibrillar aggregates and plaques) [37,38]. Recently binding of 3D6 to Aβ-oligomers from an AD animal model was shown, but could not be confirmed for CSF from AD patients [39]. Bapineuzumab is believed to mainly clear Aβ by passing the blood-brain barrier and subsequent microglial engulfment, sequestration but also peripheral sink. In Summer 2012, the sponsors of the Bapineuzumab Phase III clinical trial reported disappointing results and the discontinuation of all but one subcutaneous clinical trial [40]. In detail, the studies involving *ApoE4* carriers and non-carriers failed to show any significant benefit on cognition or functional performance, even though positive effects on the secondary biomarker endpoints (cerebral amyloid burden and CSF phospho-tau) were found. ARIA, seizures and deaths occurred more frequently [41-43]. However, by subsequent pooling of non-carrier patients with very mild AD across the studies potential treatment benefits in disability assessment score were achieved, implying that an earlier treatment in the pathogenesis might be useful.

AAB-003/PF-05236812 is a humanized 3D6 (i. e. Bapineuzumab) with mutations in the Fc domain [56] to reduce effector functions and thereby ARIAs. Therefore, an improved clinical safety profile of AAB-003 compared to Bapineuzumab could be expected. Currently two clinical Phase I trials are ongoing to evaluate the safety and the tolerability of AAB-003.

Solanezumab (m266) recognizes a linear epitope in the centre of Aβ and therefore does not bind any larger Aβ-aggregates [60]. Therefore, the only conceivable mechanisms of actions for Solanezumab are peripheral sink and sequestration. The mAb also failed some weeks later than Bapineuzumab in Phase III in two clinical trials to meet its primary cognitive and functional endpoints [61,62]. Surprisingly, in a secondary analysis a reduction in cognitive decline in very mild AD patients was observed after switching the cognition score, again implying that earlier treatment could be beneficial. There were no changes noted in biomarkers such as tau, phospho-tau, hippocampal volume, whole brain volume, or amyloid accumulation [44]. The sponsors are apparently not discouraged by the data and continue the clinical development of Solanezumab in an open-label extension study.

Gantenerumab (RO4909832 or R1450) is a fully human mAb that recognizes the N-terminal and the central region within Aβ [47,48]. The binding profile of the mAb was engineered by *in vitro* maturation on fibrillar Aβ, resulting in a mAb that binds Aβ monomers and fibrils, conformed by x-ray diffraction. In addition, it was described, that Gantenerumab neutralizes Aβ_42_ oligomers. These oligomers, however, were pre-treated with HFIP and diluted in Tris-buffer resulting in a mixture of LMWO and higher aggregates to our knowledge [28], therefore oligomer-specificity has yet not directly been shown. In a Phase I clinical trial, Gantenerumab reduced cerebral amyloid, but also ARIA were observed. The human mAb is currently in Phase III.

GSK933776 is a humanized mAb directed against the N-terminus of Aβ, believed as linear epitope [55]. The Fc domain of GSK933776 was mutated to reduce the risk for vasogenic edema. Development for AD was discontinued after Phase I in 2011.

The so-called second generation of anti-Aβ mAbs is in development to target pathogenic Aβ multimers rather than Aβ monomers or fibrils. However, as already mentioned the prefibrillar Aβ-preparations are usually not well defined making it difficult to judge the recognized pathogenic species. Moreover, since binding studies for characterization are usually performed in an ELISA-like assay with the full IgG, it is not clear whether affinity or avidity was measured.

BAN2401 is the humanized mAb158 derived from mice immunized with protofibrils derived from the arctic mutation of Aβ_42_. Arctic Aβ is not able to fibrillize and thus remains prefibrillar [63]. BAN2401 was the first mAb believed to selectively bind, neutralize and eliminate protofibrils. Nevertheless, affinity for other aggregate species than protofibrils cannot be ruled out since the primary characterization of the here applied aggregates is based on SEC data revealing a peak at the exclusion limit of the used column. This peak might comprise a broad range of aggregates larger than 60 kDa. Their ELISA data give rise for an antibody, which is rather specific for prefibrillar and fibrillar Aβ. BAN/mAb158 is being evaluated in clinical Phase II.

Crenezumab (MABT5102 or RG7412) was derived by immunization with modified Aβ_1-15_[52], containing a human IgG_4_ backbone to reduce effector function [64-66]. MABT5102A is supposed to target multiple conformational protofibrillar epitopes of Aβ, including oligomeric forms, while inhibiting aggregation and promoting disaggregation of Aβ [67]. Though, having a closer look at the preparation of the Aβ_42_ aggregates, this appears rather an empiric than a defined preparation. The only characterization method for the Aβ preparation was SDS-PAGE and controls or head-to-head comparison with other mAbs were missing in the activity assays. A Phase I clinical trial proved safety, Phase II is ongoing.

SAR228810 was derived from 13C3 by immunization with polymerized synthetic Aβ_42_ peptide while the degree of fibrillar Aβ_42_ content was monitored by circular dichroism spectroscopy [57,68]. The immunogenic peptide was thereby rather well defined. 13C3 is therefore believed to recognize a conformational epitope of prefibrillar Aβ aggregates. The humanized 13C3 is in clinical Phase I evaluation.

BIIB037/BART is a novel fully human IgG_1_ and was generated using a reverse translational medicine approach screening endogenous anti-Aβ antibodies from an AD patient with an unusual stable clinical course [58]. BART apparently shows a high affinity/avidity for insoluble fibrillar Aβ and a 100-fold decreased affinity for Aβ monomers. The applied Aβ-preparation for characterization of this antibody comprises a broad range of aggregates and the terminus fibrillar is not well defined. Thus, it is not surprising that BART reveals substantially identical affinity to monomeric and fibrillar Aβ since it is specific for any Aβ-aggregate species [69]. In APP transgenic mice BART reduced amyloid burden while Aβ plasma increase was not observed. Microglia appeared to play a pivotal role in clearing plaques [70]. A Clinical Phase I trial is currently ongoing.

Aβ_20-42_ globulomers, a condensed and hydrophobic oligomer in presence of 0.2% SDS, were used for immunization to develop the mAb A-887755 that is supposed to differentiate Aβ globulomers from all other Aβ species, especially monomers and fibrils [71,72]. But again, the characterization was based on SEC, WB, thus not allowing for a definition of the Aβ-aggregate species. Nevertheless, A-887755 has high affinity/avidity for immobilized Aβ_20-42_ globulomers and detects endogenous Aβ species but not in non-demented age-matched control patients nor in vascular Aβ deposits. A-887755 was characterized in preclinical studies and has yet not been advanced to the clinic.

To summarize, many different aggregation protocols have been applied to generate Aß-aggregates for immunization resulting in a broad range of applied Aß-species. Taking into account that the applied species have a transient nature, the fate after injection is not clear and thus the effective antigen as well not. Furthermore, the terms used for the definition of Aβ-aggregate species are still not coherent and hamper any comparison.

Currently, there is a small chance of proving the clinical efficacy of anti-Aβ mAbs in ‘ordinary’ clinical trials, since early diagnosis based on CSF and imaging biomarkers for a successful treatment with disease modifying drugs has still not been achieved. One – probably last – chance would be preventive trials in familial AD cases that were currently started with Crenezumab. Remarkably, efficacy will be tested in a five-year prevention trial in an extended Columbian family carrying an AD-related mutation, which causes early onset AD [73]. Moreover, the “Dominantly Inherited Alzheimer's Network”, DIAN, will launch clinical trials with Solanezumab and Gantenerumab soon in large families with genetic mutations that make them susceptible to the disease [45].

## Naturally anti-Aβ antibodies and IVIG treatment: is polyclonal the key?

Polyclonal naturally occurring autoantibodies against Aβ (NAbs–Aβ) are found in serum of healthy persons and are reduced in AD patients [74-76]. Intravenous immunoglobulins, i.e. IVIGs, are commercially available as polyclonal Ig preparations purified from human plasma and are authority-approved for the treatment of immunosuppression, autoimmunity and a variety of other neurological conditions. IVIG contains NAbs–Aβ and interestingly, regular IVIG treatment reduced the risk of developing AD by more than 40% in these patients [77].

Moreover, NAbs–Aβ seem to inhibit the propensity of Aβ to aggregate, thereby blocking its toxicity, and affected the clearance of Aβ, but NAbs–Aβ did not readily clear senile plaques although early fleecy-like plaques were reduced. [78]. In epitope mapping, NAbs–Aβ detected mainly the mid-/C-terminal epitope of Aβ, starting at the amino acid 28. NAbs–Aβ are believed to preferentially capture apparent dimers and trimers and interfere with oligomers [79,80], which were prepared as described from Kayed *et al.*[24]*.* It is hypothesized that NAbs–Aβ rather recognize a common conformational epitope than a distinct peptide sequence [81]. In active Aβ immunization studies in AD patients, fibrillar Aβ_42_ was used as an antigen, suggesting that N-terminal epitopes of the Aβ peptide were predominantly exposed and were available as binding sites. Accordingly, active immunization like AN-1792 generated primarily antibodies that recognized the N-terminus [82]. Likewise, Aβ peptide is deposited in a fibrillar form in the plaques, and the N-terminal part of the Aβ peptide is mostly available at the plaque surface. In contrast, NAbs–Aβ, mainly directed to the central and C-terminal epitopes of Aβ, are less capable of binding the N-terminus of Aβ; thus NAbs–Aβ do not clear plaques.

In different clinical pilot studies [76,83,84], IVIG affected plasma Aβ, improved cognition and the amount of Nabs-Aβ in patient’s serum increased dose-dependently with IVIG treatment. However, those trials did not have strong statistical significance. In a subsequent small Phase II study, IVIG exhibited a dose-dependent effect on brain atrophy [85] that was assumed to be correlated with improvement in clinical outcomes [86]. Preliminary data released in summer 2012 [49,50] showed a three-year stabilization of AD symptoms with IVIG, including no decline in cognition, memory, daily functioning and mood. The patient number involved is rather small: out of 16 patients who completed the open-label three-year follow-up, four patients receiving the most effective dose were principally unchanged from their cognitive baseline. A recently completed study using IVIGs from another manufacture has not met its primary endpoints in improving or stabilizing cognition [51]. Conversely to previous studies, no changes in plasma and CSF Aβ could have been detected during the treatment. The only apparent benefit of the treatment was a significant improvement in cerebral glucose metabolism.

Several Phase II and III trials of IVIG in AD are currently ongoing. Meanwhile, the sponsors of the largest current trials are less optimistic to achieve endpoints in mild-to-moderate AD [87]. While IVIG could apparently demonstrate a positive signal in early AD, the effects on cognition are unlikely to be statistically significant in more advanced AD. Possibly, the previous results from small cohorts included patients early in AD pathogenesis and are therefore not comparable with the studies from Bapineuzumab and Solanezumab. Finally, in geriatric patients the use of IVIG is often limited by renal insufficiency [88] and we would not have enough blood donors to supply all upcoming AD patients with IVIG [89] in case that Phase III trials give positive results.

## Conclusion

To summarize, the reported and potentially relevant Aβ-aggregates range from the smallest possible aggregate – the dimer – up to particles with hundreds of kDa. Furthermore, all of these reveal neuronal impairment in AD models. Including the many reported methods for Aβ-aggregate preparation one might question whether the whole spectrum of prefibrillar Aβ-aggregates is of relevance in AD and that some Aβ-aggregative species might share conformational motifs exerting pathophysiological effects [90]. Possibly, the ongoing polymerization process promotes Aβ-related neurotoxicity [91] via many transient aggregate intermediates. In addition, most of the described Aβ-aggregates are not covalently bound and reveal remarkable meta-stability, with the ability for reorganization within different aggregate equilibria [11,17,28]. It is conceivable that commonly used methods for Aβ characterization provide only an isolated view of individual Aβ-species as opposed to the entire spectrum of Aβ-aggregates. This also has implications for immunization with Aβ-aggregates, since their fate after injection is entirely elusive owing to their transient nature.

Therefore it might be a long run to define a species as well as to pick out the corresponding antibody. There might be a conservative conformation shared by prefibrillar aggregates, which could be identified as promising target, however this is still elusive. We assume that explicitly hunting for conformation specific monoclonal antibodies is less promising than depleting whole Aβ from the brain with a polyclonal approach unless the toxicity-mediating motif has been identified. Polyclonal IVIGs appear to recognize more than one species of Aβ, which would probably explain why patients treated with IVIGs for immunological indications have a reduced risk to develop AD.

From the regulatory point of view, the approval of a highly innovative active substance for the treatment for AD still remains a challenge. Although, biomarker strategies have been more and more taken into account, the current study designs for AD superficially address the silent pathogenesis of the disease. Hence, meta-analysis of current clinical trials confirms that preventive approaches might be the right tool to avoid the onset of AD. Furthermore the selection of a geriatric-friendly application form might play an important role with respect to a “total compliance”. Therefore, it might be the more promising strategy to approach the whole spectrum of Aβ aggregates rather than to focus on a distinct aggregative species by acute treatment, thus, to generally diminish Aβ by means of a control-released immunotherapy suitable for a geriatric population. Possibly, IVIG or the translational medicine approach can support the hunt for the one toxic species of Aβ as it seems that we have the key in our plasma, recognizing pathogenic proteins.

## Abbreviations

aa: Amino acid; Aβ: Amyloid-beta; AD: Alzheimer’s disease; AßO: Aß oligomers; ASPD: Amylospheroids; ADL: Alzheimer’s disease; ADDLs: Alzheimer derived diffusible ligands; APP: Amyloid Precursor Protein; ApoE4: ApolipoproteinE4; ARIA: Amyloid-related imaging abnormalities; CSF: Cerebrospinal fluid; DIAN: Dominantly inherited alzheimer's network; ELISA: Enzyme linked immunosorbent assay; Fc: Fragment crystallizable; HFIP: Hexafluoroisopropanol; HMW: High molecular weight; Ig: Immunoglobulins; IVIG: Intravenous immunoglobulins; LMW: Low molecular weight; NAbs–Aβ: Naturally occurring autoantibodies against Aβ; phospho-tau: Hyperphosphorylated tau; SDS-PAGE: Sodium dodecyl sulfate polyacrylamide gel electrophoresis; SEC: Size-exclusion chromatography; Tris: Tris(hydroxymethyl)aminomethane; WB: Western blotting.

## Competing interests

JM and KS are former employees of Boehringer Ingelheim Pharma, CM is a former employee of Ratiopharm GmbH. KS supported and holds a patent related to this field.

## Authors’ contribution

JM supported with novel data on Aβ conformation and aggregation, CM supported with insight from regulatory affairs and KS supported with insight on Aβ immunotherapy und neuroimmunology. All authors drafted the manuscript. All authors read and approved the final manuscript.
